# Chimpanzee adenoviral vectors as vaccines for outbreak pathogens

**DOI:** 10.1080/21645515.2017.1383575

**Published:** 2017-10-30

**Authors:** Katie Ewer, Sarah Sebastian, Alexandra J. Spencer, Sarah Gilbert, Adrian V. S. Hill, Teresa Lambe

**Affiliations:** The Jenner Institute, University of Oxford, Old Road Campus Research Building, Headington, Oxford, UK

**Keywords:** Vaccines, viral vectors, MERS Co-V, Lassa fever, Nipah

## Abstract

The 2014–15 Ebola outbreak in West Africa highlighted the potential for large disease outbreaks caused by emerging pathogens and has generated considerable focus on preparedness for future epidemics. Here we discuss drivers, strategies and practical considerations for developing vaccines against outbreak pathogens. Chimpanzee adenoviral (ChAd) vectors have been developed as vaccine candidates for multiple infectious diseases and prostate cancer. ChAd vectors are safe and induce antigen-specific cellular and humoral immunity in all age groups, as well as circumventing the problem of pre-existing immunity encountered with human Ad vectors. For these reasons, such viral vectors provide an attractive platform for stockpiling vaccines for emergency deployment in response to a threatened outbreak of an emerging pathogen. Work is already underway to develop vaccines against a number of other outbreak pathogens and we will also review progress on these approaches here, particularly for Lassa fever, Nipah and MERS.

## Progression of the vectored vaccine approach: Success of rapid clinical production and testing of Ebola and malaria vaccine vectors

Since the first documented report of the use of an engineered virus to induce a protective immune response,^1^ clinical testing of numerous potential vaccine vectors has been undertaken against a broad range of diseases. Over many years of preclinical development, a series of new vector or vaccination regimens have demonstrated improved immunogenicity: in particular, antigen-specific antibody and/or T cell responses have been increased through iterative rounds of vector vaccine development. This is well illustrated by the development of malaria vaccines against *P. falciparum* encoding the ME-TRAP antigen, where vaccine-induced T cell responses have increased from 44 IFN-γ spot-forming cells per million peripheral blood mononuclear cells (SFC) after DNA vaccination, to 850 SFC after a single vaccination with a simian adenovirus-vectored vaccine (Table 1). Importantly, viral vectors have not shown age-limitations in their use, with comparable T cell responses observed following vaccination with a modified vaccinia Ankara (MVA) vector expressing the influenza A antigens NP+M1 in healthy older adults (aged 50–60, 60–70, 80+ years) compared to a younger adult population (aged 18–55 years).^2^ In addition, age de-escalation studies of chimpanzee adenovirus 63 (ChAd63) ME-TRAP in West-African children have demonstrated potent T cell and antibody responses in immunised children as young as 1 week of age.^3,4^

**Table 1.** Comparison of cellular immune responses with different delivery methods for the same malaria antigen (ME-TRAP) at seven days after the final vaccination. Immunogenicity as measured by *ex vivo* interferon-gamma ELISPOT using the same ELISPOT method and peptide pools in the same lab.

Vector(s)	Regimen	Dose	Route	Prime-boost interval	N	Mean SFC/10^6^ PBMC (S.E.M)	Efficacy against malaria?^*^	Reference
Fowlpox (FP9)	FF	1 × 10^8^ pfu	i.d.	4 weeks	8	15 (85)	NT	^113^
DNA	DDD	500μg	i.m.	3 weeks	4	48 (20)	No	^114^
MVA	MMM	3 × 10^7^ pfu	i.d.	3 weeks	9	41 (13)	No	
DNA-MVA	DDDM	1000μg-3 × 10^7^ pfu	i.d.-i.m.	3 weeks	3	162 (112)	No	
FP9-MVA	FM	1 × 10^8^ p.f.u.-1.5 × 10^8^ pfu	i.d.-i.d.	4 weeks	5	350 (360)	No	^115^
FP9-MVA	FFM	1 × 10^8^ p.f.u.-1.5 × 10^8^ pfu	i.d.-i.d.	3 weeks	12	475 (375)	13% (no efficacy in malaria-exposed subjects)	^115,116^
ChAd63	ChAd63	5 × 10^10^ vp	i.m.	None	10	726 (189)	No	^14,117^
ChAd63-MVA	ChAd63-MVA	5 × 10^10^ vp- 2 × 10^8^ pfu	i.m.-i.d.	8 weeks	15	2646 (522)	21% (67% efficacy in malaria-exposed subjects)	

SFC, spot-forming cells. PBMC, peripheral blood mononuclear cells. S.E.M., standard error of the mean. Pfu, plaque-forming units. i.d., intradermal. i.m., intramuscular. vp., viral particles.

* Efficacy against controlled human malaria infection in malaria-naïve subjects.

The urgent need for a treatment or vaccine intervention during the West-African Ebola outbreak saw five vectored vaccines tested concurrently in Phase I trials; three non-replicating adenoviruses of different serotypes, MVA and Vesicular stomatitis virus (VSV), all encoding the ebolavirus glycoprotein (GP). All vaccines were primarily tested for their ability to induce high levels of antibodies against GP, as this correlated with protection observed in non-human primates, although cell-mediated immunity has also been shown to play a protective role with some vectors.^5,6^ While it is not straightforward to directly compare antibody levels induced by the different vectors due to the range of assays employed by different groups, responses following a single vaccination with ChAd3, Ad26 and rVSV were detectable within 28 days, with a very significant enhancement in antibody responses observed when adenoviral prime vaccinations were followed by an MVA boost.^7,8^ Humoral immunogenicity induced by various viral vectors encoding Ebolavirus (EBOV) glycoprotein is summarised in Table 2. Although initially developed as a platform for inducing T cell responses, single vaccinations with ChAd63 have demonstrated good antibody induction against malaria antigens, which could be enhanced by boosting with an MVA.^9-12^ Ad-MVA regimens induced IgG responses that were maintained for at least 180 days after immunisation.^7,13^

**Table 2.** Comparative humoral immunogenicity of viral vectors encoding Ebolavirus glycoprotein.

Viral vector	Regimen	Mean SFC/10^6^ PBMC	Protein ELISA Endpoint Titre	Protein ELISA EC_90_	Whole Virion ELISA	Neutralisation Titre	Reference
MVA	Prime only	25^*^	<100				^7^
ChAd3	Prime only	700	1493.6	469 EC_90_	752.4	14.9	^13,118^
Ad26	Prime only	103	600				^7^
Ad5	Prime only	765		1305.7 EC_90_			^119^
rVSV	Prime only		1780		920.7	22.2	^120^
MVA-Ad26	Prime-boost	880	17428.6				^7^
Ad26-MVA	Prime-boost	648	8098.9				^7^
ChAd3-MVA	Prime-boost	2068	9279.6	11970 EC_90_	9007	243.9	^13,121^

* lower limit of detection, same level as placebo vaccinated controls.

Note that the titres listed may not be strictly comparable because of some minor differences in methodology. However, the general scale of responses is informative. n.d. not detected.

**Figure 1.**
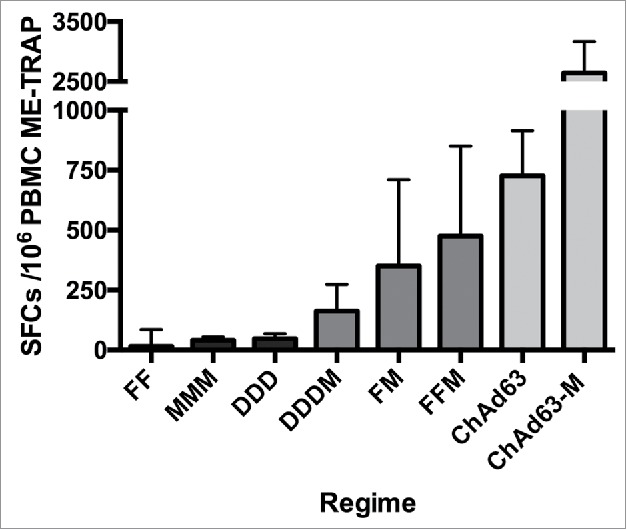
Comparative T cell immunogenicity of different viral vector regimens encoding the same pre-erythrocytic malaria antigen, ME-TRAP, as measured by *ex-vivo* interferon-γ ELISpot assays. F, Fowlpox (FP9); M, MVA; D, DNA; SFC, SFC, spot-forming cells; PBMC, peripheral blood mononuclear cells.

Prime-boost vaccination with adenoviral and MVA vectored vaccines is now well-stablished as a safe and robust strategy for inducing both cellular and humoral immunity against malaria and ebolavirus, with the addition of an MVA boost increasing both the magnitude and the breadth of the T cell response (Fig. 1 and Table 1).^13,14^ Either vaccine can act as prime or boost, as demonstrated in a novel Phase I Ebola vaccine trial with AdHu26 and the multivalent MVA BN-Filo vaccines.^7^ Although the highest T cell and antibody responses to ChAd3 MVA were observed with a four to eight-week interval between prime and boost, reducing the interval to one week still induced comparable T cell responses to the eight-week interval. However, the shorter prime-boost interval did lead to a reduction in antibody responses including neutralising antibodies.^13^

MVA vectors have been successfully used to boost responses in adults induced by vaccination with BCG in infancy, demonstrating the potential of the MVA vector to boost any pre-existing T cell memory response^15^ The development of multivalent MVA vectors, such as MVA BN-Filo which encodes four proteins from three ebolavirus species and Marburg virus, is also a potentially important tool for reducing the number of vaccine products that might need to be manufactured, by encoding protective antigens from several strains of the same pathogen or from multiple pathogens into the same vaccine construct (reviewed in^16^) The large genome of MVA allows insertion of a larger amount of foreign DNA compared with other viral vectors including adenoviruses.

Consistent with previous studies, strong T cell responses to the EBOV glycoprotein were observed after a single ChAd3 administration and significantly enhanced after an MVA boost, but were undetectable after rVSV vaccination.^17^ Only the rVSV vaccine was assessed for efficacy during the outbreak in a ring-vaccination trial where volunteers were stratified into immediate or delayed vaccination groups following exposure.^18^ The significant reduction in Ebola cases from 10 days after vaccination in this Guinea trial highlights the need to induce a rapid immune response in an outbreak scenario, with a single-dose vaccine remaining the most manageable option. For a rapid response in an outbreak setting, an early induction of protective immunity would be prioritised over durability. However, for immunisation of healthcare workers and other first responders in anticipation of a potential outbreak in the future, durability would be more important than rapid induction of immunity. For the former application, single dose vaccines will be most desirable, whereas for durable immunity a multi-dose regimen would likely be acceptable and could be required.

### Viral vector biology influences the choice of vaccine platform

Several viral vectors currently have the potential to serve as single dose vaccine platforms for the purpose of outbreak preparedness, having shown robust immunogenicity in clinical trials (reviewed in^19^). However, in order to achieve high vaccine effectiveness, it is equally important to consider parameters affected by vector biology, such as manufacturability, stability and safety of the vaccine.

A key factor is the manner in which the vaccine antigen is encoded and expressed. In adenoviral vectored vaccines, the antigen is typically placed under the control of a heterologous, strong promoter, and encoded in an independent expression cassette which is inserted into a well-characterised location in the adenoviral genome. This is most commonly the E1 locus. Concurrent deletion of the adenoviral E1 genes at this locus renders the virus replication incompetent. Vector production can therefore only take place in complementing cell lines expressing the E1 genes, such as HEK-293 or PER.C6®.^20^ Typical genetic engineering methods for antigen insertion into adenoviral vectors include plasmid-based homologous recombination in *E. coli*,^21^ bacterial artificial chromosome (BAC)-based recombineering,^22^ or *in vitro* Gateway® recombination.^23^ The placement of such antigen expression cassettes within the viral genome leads to *de-novo* expression of the antigen in the vaccine target cells, which in turn results in a strong humoral as well as cellular immune response against the antigen. More recently, the capsid-incorporation approach has shown promise for the induction of antigen-specific antibodies using modified adenoviral vectors.^24^ Here, antigenic epitopes or entire antigens are engineered to be part of adenoviral capsid proteins and are thus displayed on the surface of the viral vector, for recognition by the immune system. However, as the capsid-display strategy has not yet been evaluated in clinical trials, this review will focus on traditionally engineered adenoviral vectors with antigen cassettes at the E1 locus.

The first adenoviral vaccine vectors to be developed were based on human adenovirus serotype 5 (HAd5), a species C adenovirus which commonly infects humans. However, it was found that pre-existing anti-HAd5 antibodies which are present in a large proportion of the human population could significantly dampen the humoral and cellular immune response to the vaccine antigen.^25^ Various strategies have since been explored to circumvent this problem: the use of alternative human serotypes, such as HAd26 or HAd35,^26^ re-engineering the capsid of HAd5 to prevent antibody recognition,^27^ and the use of simian adenoviral vectors against which there is no pre-existing immunity.^28^ As discussed above, chimpanzee adenoviral vectors (ChAds) have successfully been used in clinical trials against a variety of diseases.

ChAds are non-enveloped viruses, meaning that the antigen (e.g. a membrane glycoprotein) is not present on the surface of the vector, but is expressed at high levels once the vector enters the target cells of the vaccinated individual. This is in contrast to VSV-based vaccine vectors, which, as enveloped viruses, are designed to incorporate glycoprotein antigens into their viral lipid membrane and thus display the antigen on the virus surface, in addition to expressing it upon entry into the target cell.^29^ Crucially, VSV-based vectors carrying heterologous glycoprotein antigens are generally deleted for their endogenous glycoprotein (VSV-G), which implies that it falls to the vaccine antigen to fulfil the role of functional viral fusion protein as an essential component for vector propagation during manufacture as well as for target cell entry. This important requirement for functionality inherently affects the choice of antigen for VSV-based vectors, as some viral glycoprotein antigens are either not functional by themselves (e.g. Nipah virus glycoprotein G needs glycoprotein F^30^ or are not incorporated into the VSV membrane without modification (e.g. HIV env^31^). In addition, while adenoviral vectors can equally well encode antigens which are not membrane-bound glycoproteins (e.g. Ebolavirus nucleoprotein, HIV gag), VSV vectors carrying such antigens rely on the endogenous glycoprotein (VSV-G) for viral entry. Since the full-length VSV-G protein is implicated in neurotropism,^32^ a genetically attenuated vector carrying a truncated VSV-G has been developed,^33^ which has an acceptable safety profile in healthy adults.^34^

Having thus weighed up some of the characteristics of the two most clinically advanced vectors for emergency preparedness platforms, it becomes apparent why vector biology can have significant implications for vaccine safety. Specifically, tissue tropism and replication competency of the viral vector have to be taken into consideration. Intuitively, a replication-deficient vector (such as ChAd) carries less safety risks than a replication competent, albeit attenuated, vector (such as VSV), since the inability to replicate prevents dissemination of the vector throughout the body. Accordingly, transgene expression of replication-deficient adenoviral vectors was shown to be confined to the injection site and the draining lymph nodes,^35^ whereas recent Phase I/II trials of rVSV-ZEBOV found evidence of viral vector replication in synovial fluid and skin lesions, presumed to be a result of Ebolavirus glycoprotein-specific tissue tropism of the vaccine.^8^ These findings underline the difficulty in predicting the safety profile of VSV-based vaccines, since tissue tropism will be highly dependent on the chosen glycoprotein antigen. In contrast, adenoviral vectors have a well-characterised safety profile, across a range of age groups, which is largely independent of the nature of the antigen.^36^

Lastly, vector biology may also significantly impact vaccine manufacture and delivery. For emergency preparedness stockpiling, each vaccine might need to be produced at a scale of 500,000 – 2 million doses, with the option to quickly increasing manufacture to perhaps 4–6 million doses or more in case of an outbreak, depending on the specific pathogen. Of the two most clinically advanced platforms, VSV and adenoviruses, the latter can likely meet this requirement more easily: GMP-compliant large-scale adenoviral vector production facilities exist in many countries, related to the regular use of adenoviral vectors not only in prophylactic vaccines but also in some cancer and gene-therapy trials. One potential drawback of ChAd-based viral vectors compared to human Ad vectors is the need for vector optimisation or cell line engineering to ensure high viral yields during virus production. For example, ChAd vectors may need to contain certain E4 genes from HAd5 in order to grow to high titers in current HAd5-E1-transcomplementing cell lines, as was demonstrated with the ChAdOx1 vector.^23^ Alternatively, producer cell lines can be engineered to increase viral yield.^37^ However, this need for optimisation has not been a hurdle to large-scale manufacturing so far. GMP-compliant VSV-vector production has also been developed in recent years, and scalable manufacture of rVSV is now possible.^38^ Once a stockpile has been produced, vector stability during storage and deployment is critical. Most viral vectored vaccines are stable for >5 years at −70°C, and a 2–8°C cold chain is required for distribution and storage of adenoviral vectors. One study assessing the recently deployed rVSV-ZEBOV vaccine observed a significant loss of viral titres at a temperature of 4°C after 2 weeks,^39^ whereas adenoviral vectors were shown to be stable for 20 days at room temperature in a sucrose buffer.^40^ In addition, sensitivity of any VSV-based vaccine to pH changes is presumably dependent on the specific envelope glycoprotein (i.e. the vaccine antigen). Overall, vaccine stability in terms of temperature and pH range would therefore likely be variable across a panel of putative VSV-based outbreak vaccines, since the glycoprotein will differ from vaccine to vaccine. In the case of an adenoviral vectored vaccine, on the other hand, variation in stability is expected to be minimal, since the vaccine antigen is not present in the viral capsid, and the composition of the virus particles would be very similar across different vaccines. Of note, new approaches for thermostabilisation have recently been developed for adenoviral vectors, such as immobilization of viral particles in a sugar glass on a filter^41^ or the use of biocompatible additives to slow down the degradation of virus particles.^40^ These improvements are expected to have a significant impact on the deployment of vectored vaccines in challenging climates such as sub-Saharan Africa. In human populations, pre-existing immunity to simian-derived adenoviral vectors is, unsurprisingly, less prevalent than immunity to human adenoviruses, and antibodies to some simian vectors, such as ChAdOx1, appear to be particularly rare.^23^ Anti-vector immunity to the backbone of simian viruses increased after vaccination but is relatively short-lived. As a result, reuse of the same vectors has been successful for boosting after 6 or more months in clinical trials.^42^

### Limitations of the traditional approach

The traditional approach to vectored vaccine design has been to identify an immunogenic antigen from the pathogen, construct the vector in the chosen platform and then assess immunogenicity and efficacy in murine models, prior to further testing in higher species and progression to the next stage of vaccine development. A significant obstacle in this approach is that the pathogen must be infectious in rodents if the efficacy of the vaccine is to be assessed preclinically and therefore the data may rely on mouse-adapted or chimeric pathogens (Table 4 summarises common mouse models for evaluating candidate vaccines for outbreak pathogens).

In the case of MERS CoV or SARS CoV, preclinical vaccine candidates could be tested in murine models with a mouse adapted strain of virus,^43,44^ but for newly emerging pathogens, establishing a mouse model could take significant time, in particular for evaluation of numerous viral isolates or serial passaging of a virus in mice. Alternatively, use of neonatal mice or knockout mice (e.g. of IFN-α/βR, STAT-1) have been required to mimic human disease for Ebola, Marburg, Lassa, Nipah, or Zika viruses in mice, and only through expression of human DPP4 (receptor for MERS CoV) in mouse lungs could infection of mice with MERs CoV be achieved.^45^ While these mouse models may prove useful in drug discovery, if a significant component of the immune response is compromised, it is unlikely that protection observed in pre-clinical studies will be consistent with the protective immune response required in humans.

### A new strategy for developing vaccines against outbreak pathogens

A more economical and achievable strategy than traditional approaches to vaccine development and deployment would be to focus on manufacturing small stockpiles of vaccine using a common platform technology. ChAd vectored vaccines provide a good example of a suitable vaccine platform, which has been identified as one of significant interest by the WHO R&D Blueprint process. The overall strategy would be to generate suitable stockpiles for emergency response use having previously demonstrated safety and immunogenicity of each vaccine up to Phase II trials in the target geographical regions. These products could be stored in relevant locations for each disease and, in the event of an outbreak emerging, could be deployed in a ring vaccination program similar to that employed in a Phase III trial in Guinea of the rVSV ZEBOV vaccine during the West African Ebola outbreak.^18^ Such a deployment would need to be made under the provisions of policies for use of unapproved medicinal products, such as the FDA Expanded Access program, also known as “compassionate use”, or other emergency use legislation. This would require fulfilment of certain conditions including that no comparable or satisfactory therapy is available, that the risk of harm from the vaccine is not greater than the risk of disease and that there is sufficient evidence of the safety and effectiveness of the product to support its use in the given circumstances.^46^ In this context, a vaccine for an outbreak pathogen, based on a well-developed platform, such as ChAd vectors, with evidence of efficacy from a relevant animal model would be likely to gain approval for use in a limited setting. Based on research, manufacturing and clinical trial costs for the ChAd3 vectored vaccine developed for Ebola, vaccines might be stockpiled for just $50 million per disease, representing a fraction of the cost of bringing a vaccine through to licensure. Deployment would provide the efficacy data in humans required for approval by a national regulator, increasing the likelihood of the vaccine progressing through the later stages of development.

### Tackling future outbreak threats

To improve responsiveness to epidemics, in 2015 the WHO published a list of nine diseases requiring urgent vaccine R&D to prevent public health emergencies in the future. This list was revised in 2017, and key characteristics of the diseases prioritised by the WHO are summarised in Table 3. The process of prioritising diseases took into account properties of the causative pathogen e.g. transmissibility, host-based factors such as immunopathology, clinical aspects including ease of accurate diagnosis, availability of countermeasures and mortality, public health capacity and epidemiological factors.^47^ Research and development priorities for these diseases include development of suitable diagnostic tests, assessment of potential treatments, identification of key knowledge gaps, production platforms, behavioural interventions and acceleration of vaccine development. Preparation of sufficient quantities of safe and efficacious vaccines against potential outbreak pathogens is an extremely effective strategy. However, a lack of access to dedicated long-term funding has hampered vaccine development for outbreak pathogens in recent decades.^48^ As well as limiting the number of new vaccines being developed, the number of facilities with the capacity to biomanufacture vaccines is also limited, which is a significant issue for outbreak preparedness.^49^ In addition, WHO recognised that generally applicable platform technologies for rapid vaccine development are required and have set out to identify and prioritise the leading platforms.

**Table 3.** Mouse models for evaluating candidate vaccines for outbreak pathogens.

Disease	Mouse-adapted virus strain?	Knock-out mouse line	Age of mice	Paper/Review of model
Ebola	Yes	IFN-α/β R^−/−^ or Stat1^−/−^	Neonates	^122^
Marburg	No	IFN-α/βR^−/−^	Neonates	
Lassa	No	STAT1^−/−^		^123^
Nipah	No	IFN-αR^−/−^	Adults BALB/c and C57BL/6 intranasal inoculation (Dups 2014)	^124^
CCHF	No	STAT1^−/−^		^125^
MERS	Yes			^43^
SARS	Yes			^44^
Zika	No	IFN-αR^−/−^		^126^
Chikungunya	No	IFN-α/βR^−/−^	Neonates	^127^

**Table 4.** Characteristics of the priority diseases identified in the WHO R&D blueprint (revised 2017).

Disease	Causative agent	Family and genus	Host / vector	Transmission to humans	Case fatality rate in humans	Geographical distribution
Arenaviral haemorrhagic fevers (including Lassa Fever)	Arenavirus, e.g. Lassavirus (LASV)	*Arenaviridae Mammarenavirus*	*Mastomys* rats	Contact with rat urine or faeces, contact with infected body fluids	1%	West Africa
Crimean-Congo Haemorrhagic Fever (CCHF)	Crimean-Congo Haemorrhagic Fever virus (CCHFV)	*Bunyaviridae Nairovirus*	Domestic animals/ *Hyalomma* ticks	Tick bites, contact with infected livestock at slaughter, contact with infected body fluids.	10-40%	Africa, the Balkans, Middle East, Asia
Filoviral disease- Ebolavirus disease	Ebolavirus (EBOV, SUDV, RESTV, BDBV, TAFV)	*Filoviridae Filovirus*	Fruit bat (Pteropodidae family)	Contact with infected wild animals or infected human body fluids.	25-90%	Central and West Africa
Filoviral disease- Marburg virus disease	Marburgvirus (MARV, RAVV)	*Filoviridae Marburgvirus*	Fruit bat (*Rousettus aegypti*)	Contact with bats or infected human body fluids	24-88%	Central Africa
Middle East Respiratory Syndrome (MERS)	MERS coronavirus (MERS-CoV)	*Coronaviridae Betacoronavirus*	Bats, dromedary camels	Contact with bats or camels, Human-to-human transmission common	36%	Middle East, Korea
Severe Acute Respiratory Syndrome (SARS)	SARS coronavirus (SARS-CoV)	*Coronaviridae Betacoronavirus*	Bats, palm civets	Primarily human to human through infected respiratory secretions and faeces.	9%	China, Hong Kong, Vietnam
Nipah (and related Henipaviruses) disease?	Nipah virus (NiV), also Cedar, Hendra.	*Paramyxoviridae Henipavirus*	Fruit bat (Pteropodidae family), pigs	Contact with bats and pigs	75%	Malaysia, India, Bangladesh
Rift Valley Fever	Rift valley fever virus (RVF)	*Bunyaviridae Phlebovirus*	Domestic animals, *Aedes* mosquito	Contact with infected livestock tissue, occasionally infected mosquitoes	Up to 50%^*^	Africa, Middle East
Severe fever with thrombocytopaenia syndrome (SFTS)	Severe fever with thrombocytopaenia syndrome virus (SFTSV)	*Bunyaviridae Phlebovirus*	Ticks, mites, domestic animals	Tick bites	7.3%^128^	China, Japan, Korea, USA.
Zika disease?	Zika virus (ZIKV)	*Flaviviridae Flavivirus*	*Aedes* mosquito	Infected mosquito bite	Very rare.	Africa, Asia, Micronesia, Americas

* in haemorrhagic fever cases.^47^

To address these issues, the Coalition for Epidemic Preparedness Innovations (CEPI) was launched in January 2017, bringing together funders including the Wellcome Trust, the Bill and Melinda Gates Foundation, and the governments of Norway, Germany, Japan and others.^50^ The initial fund is $460 million, with the European Commission also pledging co-funding of €250 million and further funding due to be confirmed from the Government of India by the end of 2017. The fund will initially focus on the Nipah, Lassa and MERS viruses, aiming to bring two candidate vaccines through development against each disease. CEPI also aims to promote technical and institutional platforms to improve responsiveness to future epidemics. The approach undertaken by CEPI will advance vaccine development for diseases where research to date has been limited. This is in large part due to the lack of market potential for such vaccines in conjunction with the huge costs involved over a long period of time to provide a vaccine, from pre-clinical development through to licensure, estimated at upwards of $200 million to $500 million per vaccine.^51^ Therefore, the funding required to license a vaccine for each of the priority diseases highlighted by the WHO blueprint would run into many billions of dollars, and opportunities to assess the efficacy of these vaccines in humans would be rare.

### Prioritising vaccine development for the greatest threats

Although Ebola virus disease (EVD) has been described since 1976, the outbreak that began in 2014 was larger than all the previous episodes combined, potentially due to a mutation in the glycoprotein that occurred immediately prior to the rapid increase in the number of EVD cases.^52,53^ Although not sufficiently advanced to be deployed immediately during the outbreak itself, several vaccines against ebolaviruses had already been manufactured to Good Manufacturing Practice (GMP) standards providing a rare opportunity to undertake phase I trials very rapidly and then assess efficacy against disease.

The 2014 outbreak provided a much-needed impetus to improve pandemic preparedness for emerging pathogens. To this end, the three identified viruses as targets for vaccine development, by CEPI have known potential to cause outbreaks with high mortality: MERS-CoV, Nipah virus and Lassa virus.

### Nipah virus

Nipah Virus (NiV) is a recently-recognised and highly pathogenic zoonotic paramyxovirus that can cause severe disease in man with high associated fatality rates (up to 100%).^54^ Outbreaks have occurred in Malaysia, Singapore and India with almost annual occurrence in Bangladesh. Human-to-human transmission is common in Bangladesh and has also been documented in India.^55^ Several species of pteropid fruit bats are known to be host reservoirs of NiV, with accumulating evidence that both NiV and other paramyxoviruses can circulate worldwide in bats.^54-56^ The high fatality rate, direct infection from natural reservoirs, infection following amplification in susceptible domestic livestock such as pigs, documented human-to-human transmission, and the potential ability to transverse the globe, all emphasise the pandemic potential of NiV.^56^

There are no clinically approved vaccines against NiV, however, one therapeutic approach (monoclonal antibody therapy) has recently completed a phase I clinical trial with results still to be reported.^57^ While monoclonal antibody treatment may be efficacious in a short window post-exposure, this treatment option is not suitable for large-scale use, and as such, vaccine development is a key research focus for the prevention of NiV-mediated disease. Advantageously, there are a number of animal models of NiV infection which are used in vaccine development programs and are considered to sufficiently mirror NiV-induced pathogenesis observed in humans, e.g. the hamster, ferret and African Green Monkey (AGM) models.^58-60^

While vaccine-mediated cellular immunity has been demonstrated to play a role in protection in preclinical models of NiV infection,^61^ the most advanced vaccine modalities demonstrating clear efficacy across multiple animal models have primarily induced humoral immunity. A soluble glycoprotein (sG) subunit vaccine from the related henipavirus Hendra virus (HeV) is an extensively studied vaccine that can protect ferrets and AGM from experimental challenge with NiV or HeV. Prime-boost regimens with adjuvanted HeV-sG subunit proteins are efficacious in stringent NiV challenge models, across a range of doses (4–100ug), and with pre-challenge neutralising antibody titres as low as 1:28.^62,63^ The HeV sG vaccine (Equivac® HeV) has been licensed to vaccinate horses in Australia against HeV.^64^ A number of viral vectored vaccines have also been tested and show promising immunogenicity and/or efficacy against NiV-mediated disease. These include poxvirus (canarypoxvirus ALVAC strain), vesicular stomatitis virus (VSV), rabies virus (RABV), adeno-associated virus (AAV), Newcastle disease virus (NDV) and Venezuelan equine encephalitis virus (VEEV); this topic has recently been comprehensively reviewed.^56,65^

### Lassa virus

Lassa virus (LASV) is a medically relevant arenavirus which produces conditions ranging from asymptomatic infection to a lethal haemorrhagic fever, Lassa fever (LF). Annually, LASV appears to infect between 300,000 to 500,000 individuals with mortality rates ranging from 2% to in excess of 50% in outbreaks.^66,67^ LF is an endemic zoonosis in parts of West Africa including Nigeria, Liberia, Sierra Leone and Guinea, with more recent studies highlighting the spread of LASV into surrounding areas e.g. Mali, Benin and Ghana. This epidemiology suggests that efficacy trials of Lassa fever vaccines could be conducted successfully in countries such as Nigeria and Sierra Leone.

The common African rat (*Mastomys natalensis)* is the zoonotic reservoir for LASV and is thought to facilitate the ease of LASV spread to humans. Despite the recurrent and high disease incidence with associated significant morbidity and mortality, there are no approved vaccines. Currently, LF treatment relies on supportive care and, where available, the administration of the antiviral drug ribavirin.^68^ There continues to be an unmet need for medical interventions that can curb the spread of LASV and avert the morbidity and mortality associated with potential viral dissemination into a large geographical area due to the zoonotic reservoir.^69,70^

The first clinically available vaccine for the prevention of an arenavirus haemorrhagic fever was Candid #1, a live-attenuated vaccine against Junin virus infection, available through the Argentine National Immunization Plan.^71^ Unfortunately, the development of a LASV vaccine has not progressed as rapidly. Cellular immunity is thought to be critical for survival of LF infection, with early T cell activation associated with a better clinical outcome.^72,73^ Recent studies focusing on the early stages of LF in non-human primates (NHP) have confirmed previous observations that early and strong T-cell responses are associated with effective control of virus replication and recovery, while fatal LASV infection of NHP has been associated with a lack of peripheral T-cell activation.^73,74^ It has also been demonstrated that some vaccination strategies primarily aimed to elicit LASV-specific humoral immunity are not effective, e.g. gamma-irradiated LASV.^75^

The development of LASV vaccines has involved a number of different platform technologies including non-replicating vaccine approaches, such as inactivated LASV virus, virus-like particles (VLPs), and DNA vaccines, as well as replication-competent vaccine strategies (both recombinant and re-assortment viral vectored vaccines). The four replication-competent LASV vaccine candidates that have been extensively studied are based on vaccinia virus,^76,77^ vesicular stomatitis virus,^78^ Mopeia virus (MOPV)^79^ and yellow fever virus (YFV) 17D vectors^80^ with all of these vaccine candidates tested in different animal models, including NHPs.

Efficacy testing in animal models that mimic the major pathophysiological and immunological features of human LF are a prerequisite before licensure. Rodents are an obvious first species to establish immunogenicity, but as LASV has a rodent host reservoir and the response to LASV varies depending on mouse strain, age and inoculation route, rodents are not suitable as a valid LF disease model. Guinea pigs are the most sensitive model to study lung pathology,^81,82^ while common marmosets (CM) are surrogates to study liver involvement.^83^ However, LASV-infected rhesus and cynomolgus monkeys are considered the gold-standard models and are the only available and relevant challenge models for human LF.

The YFV vaccine strain 17D has been genetically manipulated to express the LASV glycoprotein and was designed to control both diseases, YF and LF, in areas of overlapping incidence in West Africa.^84^ While it can protect guinea pigs,^80^ it has failed to protect marmosets and is genetically unstable.^86,87^ In addition, while recombinant vesicular stomatitis virus (rVSV) expressing LASV glycoprotein was protective in nonhuman primate challenge, the protection was not sterile and LASV viremia could be measured post-infection.^85^

LASV and MOPV are closely related Old World arenaviruses that can exchange genomic segments (reassort) during coinfection. Clone ML29, encodes the major antigens of LASV and also MOPV antigens. Preclinically, both marmosets and guinea pigs have survived an otherwise fatal LASV infection.^86,87^ Recent studies have demonstrated that SIV-infected rhesus macaques respond well to ML29 vaccination, and survive when challenged with a heterologous lethal arenavirus strain (LCMV-WE) indicating that ML29 is both safe and immunogenic in immuno-compromised animals.^88^

Another vaccine vector that proved effective in guinea pigs against LASV challenge is a Venezuelan equine encephalitis virus (rVEE) replicon particle expressing GP or NP.^89^ Animals were fully protected against LASV challenge after prime/boost/boost immunization with this vector. One of the most promising vaccines is vaccinia virus encoding LASV glycoprotein; nonhuman primates vaccinated with this vaccine candidate were protected against challenge.^90,91^ However, despite several promising vaccine candidates in pre-clinical evaluation, none has yet advanced to a clinical trial in humans.

### Novel coronaviruses: MERS CoV and SARS CoV

Several novel coronaviruses have emerged over the last decade, causing outbreaks mainly in the Middle East region and Asia, in Saudi Arabia, Jordan, Qatar and China in particular. An epidemic of Severe Acute Respiratory Syndrome (SARS) was reported in 2003, which started in China and caused over 8000 cases with between 10 and 50% mortality depending on age.^92^ The causative agent was identified as a novel coronavirus, SARS CoV, not previously identified as infectious to humans,^93^ with bats and civets as natural reservoirs.^94,95^ Middle Eastern Respiratory Syndrome (MERS) was first reported in 2012 in a man who became ill in Saudi Arabia.^96^ The isolation of another novel coronavirus followed, known as MERS CoV, which has subsequently caused nearly 1900 cases and 670 deaths.^97^ Dromedary camels are a reservoir, although transmission also occurs from human to human.^98^

Strategies for producing effective coronavirus vaccines have focussed on expression of either the spike protein or nucleocapsid proteins or, in some cases a combination of both, in a range of vectors including rabies viruses, VSV and VEE (reviewed in^99,100^). A report from a recent workshop in Riyadh on countermeasures for MERS CoV bringing together funders, public health experts and researchers concluded that progress with vaccine development is still hindered by the lack of animal models for evaluating efficacy.^100^ Small animals do not naturally express a functional form of the dipeptidyl peptidase 4 (DPP4) receptor; however, transgenic mice expressing human DPP4 are susceptible to infection.^101,102^ Despite this advance, mouse models are likely to be less useful for the assessment of immune correlates than larger animal models such as rhesus macaques and common marmosets, which exhibit the severe clinical syndromes observed in humans.^103,104^ MVA and ChAd viral vectors for MERS have reached GMP manufacture, while a DNA vaccine is now being tested in clinical trials.^105,106^

### Progress with development of chimpanzee adenovirus vectors for outbreak pathogens

In May 2017, the first cases in an outbreak of EVD were reported in the Bas Uele Province in the Democratic Republic of the Congo (DRC).^107^ This area shares a border with the Central African Republic and is particularly remote and difficult to access. As the causative species has been identified as *Zaire ebolavirus*, the rVSV-ZEBOV vaccine is being considered at the time of writing, for deployment in a ring vaccination design to protect contacts and frontline healthcare workers (HCWs).^108^ This fresh outbreak is the 8^th^ to occur in the DRC and highlights the potential utility of vaccination to protect HCWs, particularly where remote locations present significant logistical challenges for responding to and containing outbreaks. Maintaining the current momentum for developing vaccines against outbreak pathogens is crucial, and as such, simian adenoviruses are uniquely fit for purpose as an effective vaccine platform, not in small part due to their predictable safety profile, stability, manufacturability, but most importantly owing to their immunogenicity. Therefore, a single-antigen pathogen-specific ChAd vector vaccine could be suitable as a single dose approach for rapid induction of protective immunity in an outbreak, but for durable protection for potential first responders a ChAd prime, MVA boost approach could be more effective.

Novel vaccines against outbreak pathogens are under development in a range of simian adenovirus serotypes including ChAd3, ChAd63 and ChAdOx1 (reviewed in^109^) and for the human vectors AdHu26 and AdHu5. Application of a pipeline approach to developing vaccines for outbreak pathogens can greatly accelerate the output of candidate vaccines as the key processes, such as generation of constructs, production of virus stocks, defining preclinical immunogenicity, and GMP manufacture can be substantially standardized. An approach that is currently being adopted for at least twelve potential outbreak pathogens using standardized preclinical processes (Table 5), with several advancing to GMP manufacture and clinical testing. The latter include vaccines against MERS-CoV, Rift Valley fever virus, Zika virus and Chikungunya virus.

**Table 5.** Status of chimpanzee adenovirus vector (ChAd) vaccine development for a range of outbreak pathogens at the Jenner Institute, University of Oxford (as May 2017). The genetic background for all vectors is ChAdOx1 (a species E modified chimpanzee adenovirus based on isolate Y25).^23^ Antigens are inserted at the E1 locus via Gateway® recombination. For preclinical immunogenicity testing, mice typically receive a single-dose of 10^8^ infectious units (intramuscular).

Pathogen	ChAd construct made	Immunogenicity demonstrated in mice	Neutralising antibody activity demonstrated	Animal efficacy demonstrated	GMP production funded	Phase I/II evaluation commenced
Pandemic Influenza virus	✓	✓	✓	✓	✓	✓
Rift Valley Fever virus	✓	✓	✓	✓	✓	
MERS CoV	✓	✓	✓	✓	✓	
Zika virus	✓	✓		✓	✓	
Chikungunya virus	✓	✓	✓		✓	
Crimean Congo Haemorrhagic Fever virus	✓	✓				
Lassa virus	✓	✓				
*Zaire ebolavirus*	✓	✓				
*Sudan ebolavirus*	✓	✓				
*Zaire + Sudan ebolavirus* + Marburg	✓	✓				
*Yersinia pestis*	✓	✓				
Nipah virus	✓	✓				
SARS CoV	✓	✓				

GMP, good-manufacturing practice.

The key bottlenecks for this approach are the identification of vaccine antigens and the availability of appropriate animal models of disease. For preparations to be made to counter future threats, some knowledge of emerging pathogens is required, and yet detailed epidemiological surveillance for many infectious diseases remains limited in regions where incidence is greatest.^110^ Recent data suggests that around 60% of emerging infectious diseases are zoonotic with the majority originating in wildlife, requiring surveillance among livestock animals and wildlife species, as well as in humans.^111^ Although Ebola outbreaks have occurred sporadically since 1976, the pace of vaccine development for Ebola has been slow with most vaccines undergoing preclinical evaluation for more than 5 years before the start of Phase I clinical trials. The 2014–15 outbreak provided much needed momentum for public health experts and the research community to improve preparedness for future epidemics.^112^ In order to continue to improve our preparedness for future outbreaks, epidemiological surveillance and vaccine development will need to accelerate substantially.
